# RTH-149 Cell Line, a Useful Tool to Decipher Molecular Mechanisms Related to Fish Nutrition

**DOI:** 10.3390/cells9081754

**Published:** 2020-07-22

**Authors:** Guillaume Morin, Karine Pinel, Karine Dias, Iban Seiliez, Florian Beaumatin

**Affiliations:** Université de Pau et des Pays de l’Adour, E2S UPPA, INRAE, NUMEA, 64310 Saint-Pée-sur-Nivelle, France; guillaume.morin@inrae.fr (G.M.); karine.pinel@inrae.fr (K.P.); karine.dias@inrae.fr (K.D.); iban.seiliez@inrae.fr (I.S.)

**Keywords:** aquaculture, rainbow trout, RTH-149 cell line, nutrition, amino acids, autophagy, mTOR, GCN2 pathway, cell homeostasis

## Abstract

Nowadays, aquaculture provides more than 50% of fish consumed worldwide but faces new issues that challenge its sustainability. One of them relies on the replacement of fish meal (FM) in aquaculture feeds by other protein sources without deeply affecting the whole organism’s homeostasis. Multiple strategies have already been tested using in vivo approaches, but they hardly managed to cope with the multifactorial problems related to the complexities of fish biology together with new feed formulations. In this context, rainbow trout (RT) is particularly concerned by these problems, since, as a carnivorous fish, dietary proteins provide the amino acids required to supply most of its energetic metabolism. Surprisingly, we noticed that in vitro approaches considering RT cell lines as models to study RT amino acid metabolism were never previously used. Therefore, we decided to investigate if, and how, three major pathways described, in other species, to be regulated by amino acid and to control cellular homeostasis were functional in a RT cell line called RTH-149—namely, the mechanistic Target Of Rapamycin (mTOR), autophagy and the general control nonderepressible 2 (GCN2) pathways. Our results not only demonstrated that these three pathways were functional in RTH-149 cells, but they also highlighted some RT specificities with respect to the time response, amino acid dependencies and the activation levels of their downstream targets. Altogether, this article demonstrated, for the first time, that RT cell lines could represent an interesting alternative of in vivo experimentations for the study of fish nutrition-related questions.

## 1. Introduction

By 2050, the global population is expected to reach an unprecedented and alarming density. Among concerns raised by this increase, solutions need to be found to feed more than nine billion humans with affordable and nutritious foods produced with respect to the environment [1]. In this context, many expectations are placed in the aquaculture field. Indeed, aquaculture provides already more than half of the fish for human consumption every year, contributing to feeding a global population with a food widely recognized for its benefits in health. Nonetheless, new economic and ecological constraints jeopardize the sustainability of aquaculture fish production. In fact, the scarcity and the expensiveness of raw ingredients used to feed farmed fish—namely, fish meal (FM) and fish oil (FO), no longer allow affordable livestock farm fish activity [2]. Thus, one of the most important objectives of this field is to replace FM and FO from fish feed by other protein and lipid sources. So far, multiple alternatives are evaluated (e.g., plant, yeast or insect-based diet formulations) for their properties to preserve fish growth performances together with the quality of fish products [3,4,5]. To date, none of these strategies have fulfilled all the criteria required when a total replacement of FM and FO is considered. To better understand the outcomes of these replacements, numerous studies were performed over the last decades showing the decreases of feed efficiencies and/or feed intakes together with their related physiological dysregulations according to the diet formulations and fish species considered [6]. From now on, a comprehensive understanding of the molecular mechanisms dysregulated by these new diets is deeply required (1) to identify molecular and cellular markers that reflect the nutritive state of the fish when fed with specific ingredients and (2) to understand which metabolic pathway is essential for the cell growth and proliferation in fish of agronomic interests. This statement is particularly true when considering rainbow trout (RT) aquaculture. Indeed, RT, a carnivorous strict fish from the Salmonidae family, is described to have a metabolism that essentially relies on amino acids, brought until now by proteins contained in FM. Thus, due to FM replacement, farmed RT now see their growth performances considerably affected if no FM is supplied in their diets, as exemplified by studies using meals formulated with 100% of plant protein sources [7]. If some hypotheses are proposed to explain the growth retardation observed—among which, an unbalanced amino acid composition of new protein sources—evidences, notably at molecular levels, a lack to turn these hypotheses into principles.

Cell culture, an approach raised more than 70 years ago, is widely used in laboratories and certainly covers most topics in biology. If a primary cell culture was initially developed, rapidly, cell lines were established from different species [8,9,10]. The relative handling ease of cell lines combined with the reduction of animal experimentations, in accordance with the three R rules (for replacement, reduction and refinement) proposed by Russell and Burch in 1959 [11], prompted scientists to use these living biological materials as new models to study the interactions between their processes of interest and the cellular environmental conditions. Since these pioneering works, the collection of cell lines has constantly grown, in quantity and variety, to reach more than 100,000 cell lines established to date [12]. Surprisingly, and knowing that ray-finned fish represent almost 50% of the vertebrate species, only 700 fish cell lines have been established so far, with RTG-2 cells, a gonadal-derived RT cell line, as the first fish cell line [13]. Currently, the RT cell line collection, also recently called “RT Invitromatic” [14], represents almost 8% of fish cell lines generated so far and is daily used to address questions in various research fields, with the exception of nutrition. Indeed, to our knowledge, none of the studies performed so far with RT cell lines aim to decipher the molecular mechanisms related to nutrition, and the very existence, as well as the function, in these cells of the main nutrient-sensing signaling routes is still unknown. Of these, three pathways, described to be regulated by amino acids to maintain the cellular homeostasis in various species, deserve particular attention in the context of RT nutrition, which strongly relies on amino acid supplies: the general control nonderepressible 2 (GCN2), macroautophagy (hereafter referred to as autophagy) and the mechanistic Target Of Rapamycin (mTOR) pathways.

mTOR is a serine/threonine kinase described to form a multimeric protein complex called mTORC1 that is specifically activated by nutrients such as amino acids (for review, [15]). Indeed, at least four amino acids have been shown to specifically induce mTOR activation: two essential amino acids (EAA)—namely, leucine and methionine—and two conditionally essential amino acids (glutamine and arginine). Once activated, mTORC1 transduces an anabolic signal by phosphorylating its downstream targets, among which are found the Eukaryotic translation initiation factor 4E-binding protein 1 (4EBP1) and the ribosomal protein S6 kinase beta-1 (S6K) [16], which stimulates cell growth and proliferation. Moreover, through its activity, mTORC1 does not solely regulate anabolism, but it also represses catabolism, since it inhibits one of the most important intracellular degradation pathways, called autophagy. Indeed, upon starvation conditions, mTORC1 gets inactive and can no longer inhibit the initiation step of autophagy [17]. Thus, cytoplasmic materials are engulfed in double-membrane vesicles called autophagosomes. Then, autophagosomes fuse with lysosomes to degrade and recycle their contents, providing new intracellular substrates essential to cope longer with starvation. This whole process, genetically controlled by AuTophaGy-related (ATG) genes, has been shown to play a crucial role in keeping cellular and organism homeostasis, while its dysregulation, notably by unbalanced dietaries, leads to pathological disorders [18]. Finally, amino acid starvation is also known to promote the activation of the integrated stress response (ISR) pathway [19], mainly through the activation of the GCN2 kinase and, to a lesser extent, via the activation of the unfolded protein response (UPR) pathway. In both cases, the depletion of amino acids leads to the eukaryotic initiation factor 2 α (eIF2α)-dependent activating transcription factor 4 (ATF4) activation. This process represses the general protein synthesis and promotes the transcription of genes related to amino acid biosynthesis and catabolic pathways, including *ddit3* for DNA damage inducible transcript 3 (also commonly called, and hereafter referred to as, *chop* for the C/EBP homology protein), *asns* (Asparagine synthetase), *map1lc3b* (microtubule associated protein 1 light chain 3β (hereafter referred to as *lc3*) and *sqstm1* (sequestosome 1, also known as *p62*) autophagy genes.

Thus, and prior to assessing the outcomes of the use of specific nutrients on the metabolism of the RT cell lines, it appeared essential to characterize, as described in other species, these three pathways for (1) their capacity to be induced or repressed by starvation, (2) their related kinetics and (3) their specific regulation by amino acid availabilities. To do so, a series of experimentations were conducted on the RTH-149 cell line, a RT hepatoma-derived cell line studied for decades [20] in various topics. Moreover, the RTH-149 cell line seems particularly adapted to studies related to nutrition, since liver is described as a highly metabolic organ with the highest amino acid consumption rate.

In this work, we demonstrate that RTH-149 cells display functional GCN2, autophagy and mTOR pathways. Moreover, we assessed the time course of the activation of each pathway to identify the key time points at which the molecular markers implicated in these pathways reflected the most their activation states. Finally, this study opens up exciting new perspectives for the use of this cellular model by describing, for the first time, the usefulness of the RTH-149 cell line as a relevant model to address nutrition-related questions and to study the nutrient sensing signaling pathways from a comparative and evolutionary aspect.

## 2. Materials and Methods

### 2.1. Cell Culture and Treatments

RTH-149 rainbow trout hepatoma cells (ATCC^®^ CRL-1710, LGC standards, Molsheim, France) were grown at 18 °C and pH 7.4 in minimum essential medium (MEM) containing l-glutamine (#61100-053) supplemented with the MEM nonessential amino acid (NEAA) solution (#11140-50), 10% fetal bovine serum (#10270-106), 10-mM sodium pyruvate (#11360-070), 100-units/mL penicillin and 100-µg/mL streptomycin (#14065-056), all provided by Gibco (Thermo Fisher scientific, Waltham, MA, USA), and 25-mM hepes (#BP299-1, Fisher Bioreagents, Fisher scientific SAS, Illkirch Graffenstaden, France). This complete medium (CM), hereafter also referred to the control condition (CTRL), was replaced twice a week, and cells were passaged at 80–90% of confluence. Prior to experiments, cells were counted with a Cellometer K2 (Nexcelom Bioscience LLC, Lawrence, MA, USA) and plated in 6-cm-diameter dishes at a density of 400,000 cells per dish for Western blot analysis and 500,000 for real-time quantitative PCR (RT-qPCR) analysis before incubation at 18 °C. After 2 days, cells were washed twice with PBS prior treatments, according to experiments. For RT-qPCR analysis, Hank’s balanced salt solution (HBSS) (#14025-092, Gibco) supplemented with 25-mM hepes was used as the starvation medium. Amino acid treatment was composed of starvation medium supplemented (AA+) or not (AA−) with the MEM amino acid solution (#11130-036, Gibco) containing all essential amino acids (EAA), the MEM NEAA solution (#11140-050, Gibco) and l-glutamine (#250030-081, Gibco). For Western blot analysis, the starvation medium defined above was supplemented with EAA or NEAA and l-glutamine or all three (AA). Halofuginone hydrobromide (HF) (#32481, Sigma-Aldrich, Darmstadt, Germany) [21], an inhibitor of prolyl tRNA synthetase, was added to the CM to induce the GCN2 pathway at 10, 100 or 1000 nM where indicated. Treatment times were chosen in accordance with the different kinetic experiments presented hereafter. Hence, for Western blot analysis of the mTOR pathway, cells were starved for 3 h and treated by amino acids for 1.5 or 2 h. Besides the time course experiments, all RT-qPCR analyses were performed using cells treated for 24 h.

### 2.2. Protein Extraction and Western Blot Analysis

Cells were washed twice in ice-cold PBS and lysed using RIPA buffer (#89901, Thermo Scientific) supplemented with Halt protease and a phosphatase inhibitor cocktail (#78442, Thermo Scientific). Protein samples were conserved on ice for 30 min before centrifugation at 12,000× *g* at 4 °C. The concentration of protein samples was determined using the Bicinchoninic Acid Kit (#BCA1-1KT, Sigma-Aldrich). Protein samples were mixed with Laemmli buffer and subjected to sodium dodecyl sulfate polyacrylamide gel electrophoresis (SDS-PAGE), transferred on polyvinylidene fluoride (PVDF) membranes (#IPFL00010, Merk Millipore, Burlington, MA, USA) and, finally, immunoblotted using the following antibodies: anti-ribosomal protein S6 (#2217; Cell Signaling Technologies, Danvers, MA, USA), anti-phospho-S6 (Ser235/Ser236, #4856; Cell Signaling Technologies), anti-4EBP1 (#9452; Cell Signaling Technologies), anti-phospho-4EBP1 (Thr37/Thr46, #9459; Cell Signaling Technologies), anti- microtubule-associated proteins 1A/1B light chain 3B (LC3B) (#2775; Cell Signaling Technologies) and anti-β-tubulin (#2146; Cell Signaling Technologies). Membranes were incubated with IRDye secondary antibody (#926-68071, LI-COR, Inc., Lincoln, NE, USA) after washes. β-tubulin was used as the loading control and was, as for total proteins, immunoblotted after membrane stripping. Signal acquisition was performed by infrared fluorescence with the Odyssey^®^ Imaging System (LI-COR, Inc.) and quantified using ImageJ software (NIH, Bethesda, MD, USA).

### 2.3. Autophagy Flux Assay

Autophagy flux assay is one of the gold standard technics developed to assess autophagy [22]. Briefly, this technic relies on LC3, a cytosolic protein (LC3-I) that, upon autophagy induction, is conjugated to phosphatidylethanolamine (LC3-II). This conversion, described to be necessary for autophagosome formation, is used to measure the induction of autophagy in the presence of lysosomal inhibitors to prevent LC3-II degradation by hydrolases. The autophagy flux assay was therefore estimated by measuring the amount of LC3-II detected by Western blot in the presence or absence of a lysosomal inhibitor. The more the LC3-II level increases in the presence of the inhibitor, the higher the autophagy flux is supposed to be. Therefore, when indicated, cells were treated for the indicated times and media in the presence or absence of 10 µM chloroquine (CQ) (#C6628, Sigma-Aldrich) prior to proceeding to the protein extraction and Western blots analysis directed against the LC3B protein and tubulin as a loading control. Quantifications of LC3-II/tubulin ratios were then normalized to the ratio corresponding to the time point showing the highest autophagy flux (to arbitrarily define a 100% autophagy flux induction) according to the treatment considered.

### 2.4. RNA Extraction and RT-qPCR Analyses

Cells were washed twice with PBS prior to RNA extraction and purification using a RNeasy Mini Kit (Qiagen, Hilden, Germany) following the manufacturer’s protocol and stored at −80 °C. RNA concentration and integrity were determined using a Nanodrop^®^ ND1000 spectrophotometer. cDNAs were synthesized from 1 µg of RNA samples using Superscript III RNAseH -reversetranscriptase kit (#18080-093, Invitrogen, Carlsbad, CA, USA) with random hexamers (#C1101, Promega, Madison, WI, USA) according to the manufacturer’s instructions. After a first step of denaturation (5 min at 65 °C), the retro-transcriptions of RNAs were performed (5 min at 25 °C, 1 h at 55 °C and inactivation during 15 min at 70 °C) using a thermocycler. The real-time quantitative PCR reactions were performed in triplicate, comprising 3-µL Light Cycler 480 SYBR^®^ Green 1 Master, 0.76 µL of nuclease-free water (P1195, Promega), 2 µL of diluted cDNA at 1/40 or 1/80 and 0.12 µL of gene-specific primer (10 µM) (listed in Table 1) on the Roche Light Cycler 480 system (Roche, Bâle, Switzerland). Primers used for RT-qPCR analyses, validated in previous studies, are listed in Table 1 [23,24,25,26,27]. The RT-qPCR protocol was initiated at 95 °C for 10 min, followed by 45 cycles of a three-step amplification program (15 s at 95 °C, 10 s at 60 °C and 15 s at 72 °C). Melting curves were systematically monitored at the end of the last amplification cycle to confirm the specificity of the amplification reaction. Each PCR assay included negative controls (RT- and cDNA-free samples). Elongation factor 1α (*ef1)* was used for the normalization, as it was found to be the most constant and accurate across the different housekeeping genes tested (*actin, gapdh* and *18S*) and not significantly different among all the conditions tested (data not shown). The gene expression levels were presented as the relative quotient (RQ) calculated using the ΔΔCT method.

### 2.5. Statistical Analysis

All presented values are means ± S.E.M. In each figure legend is indicated as the number of biological replicates (N). Normality was tested with the Shapiro-Wilk test for each condition independently (*p* > 0.05). Then, they were analyzed using a Student’s *t*-test or one-way ANOVA, with multiple comparisons performed with Tukey’s post-hoc test or with Dunnett’s post-hoc test when comparing conditions to the control. The test applied is mentioned in each figure legend.

## 3. Results

### 3.1. The GCN 2 Pathway

**Figure 1:**
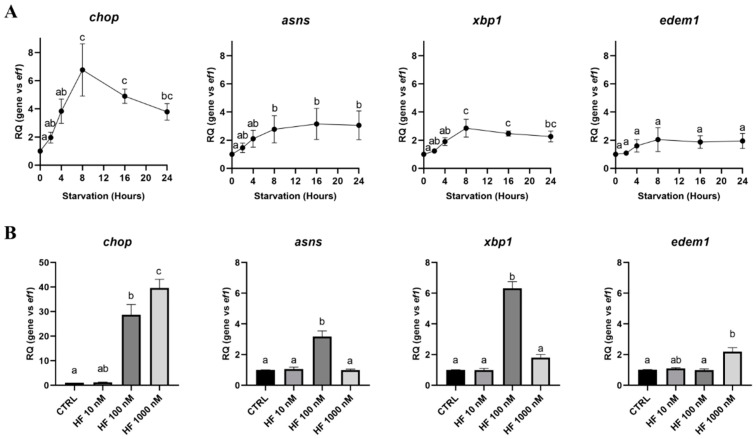
Expression of the integrated stress response (ISR) pathway target genes induced by starvation and halofuginone hydrobromide (HF) in RTH-149 cells. (**A**) Cells were starved for the indicated times prior to proceed to RNA extraction and real-time quantitative PCR (RT-qPCR) analysis of the ISR target genes. (**B**) Cells were grown in the control condition (CTRL) supplemented or not with the indicated concentration of HF. RNA was extracted 24 h following the treatments, and a RT-qPCR analysis of the ISR target genes was performed. Results are presented as the relative quotient (RQ) normalized to the *T* = 0 h time point (**A**) or control condition (**B**). (**A**,**B**) Data are presented as means ± SEM, *N* = 4. Conditions showing results statistically different from each other are presented using a different letter (*p* < 0.05, one-way ANOVA Tukey’s post-hoc test).

As mentioned previously, the GCN2 pathway is part of the integrated stress response (ISR) pathway induced following endoplasmic reticulum (ER) stress. Numerous stresses can activate ISR—among which, amino acid deficiency is certainly one of the most characterized. Therefore, we sought to evaluate if RTH-149 cells are prone to activate this pathway upon amino acid and serum deprivation, hereafter referred to as starvation, by assessing the time course of the gene expression described to be transcriptionally regulated by the ISR (Figure 1A).

**Figure 2:**
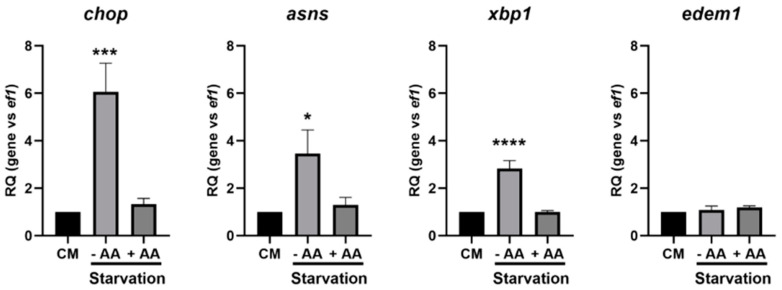
Amino acid-dependent regulation of the starvation-induced ISR gene overexpressions in RTH-149 cells. Cells were grown for 24 h in either complete medium (CM) or starvation medium supplemented (+AA) or not (−AA) with amino acids prior to proceed to RNA extraction and a RT-qPCR analysis of the ISR target genes. Results are presented as the relative quotient (RQ) normalized to the CM. Data are presented as mean values ± SEM, *N* = 3. Conditions showing results statistically different from the control condition (CM) are presented as *: *p* < 0.05, ***: *p* < 0.001 and ****: *p* < 0.0001, one-way ANOVA Dunnett’s post-hoc test.

Interestingly, we noticed that the expression of the *chop* (*ddit3)*, *asns* and *xbp1* genes reached a maximum following 8 h of starvation and remained upregulated until 24 h, while *edem1*, a target of the IRE1-activated form of Xbp1 (Xbp1 spliced variant) remained constant all along the starvation. To further characterize this pathway, HF, a pharmacological activator of the GCN2 pathway, was used to mimic amino acid starvation. The use of HF, at different concentrations in RTH-149 cells grown in the CTRL condition, confirmed the involvement of Gcn2 in the specific regulation of the genes previously observed upon starvation (Figure 1B). We observed that 100 nM of HF were sufficient to upregulate the *chop*, *asns* and *xbp1* genes. Interestingly, and later discussed, when the HF concentration was increased (1000 nM), *asns* and *xbp1* upregulations were no longer observed, while *chop* was further upregulated. Finally, we confirmed the amino acid-specific regulation of the *chop, asns* and *xbp1* genes observed upon starvation by assessing the mRNA levels of these genes in cells grown in CM or starvation conditions supplemented or not with amino acids (Figure 2).

The gene expression analysis clearly revealed that starvation-induced *chop, asns and xbp1* upregulations were directly linked to amino acid availability, since no upregulation was observed for those genes when the amino acids were supplemented in the starvation condition. Beside that these results show the conservation of the GCN2 pathway in RTH-149 cells, they also demonstrate that the GCN2 pathway is canonically activated by amino acid withdrawal.

### 3.2. Autophagy

**Figure 3:**
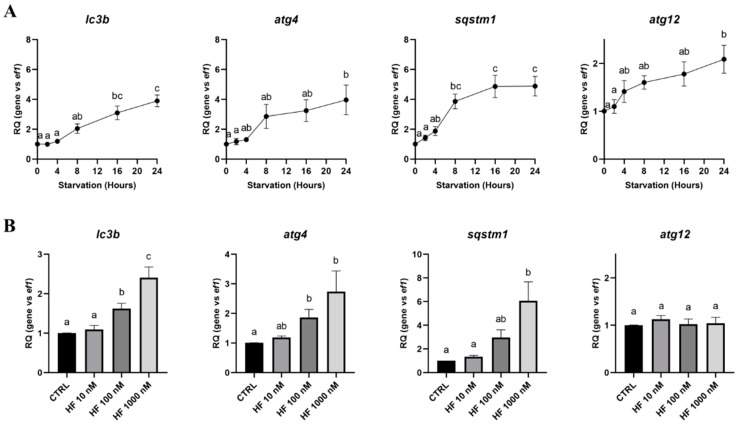
Expression of the atg genes induced by starvation and HF in RTH-149 cells. (**A**) Cells were starved during the indicated times prior to proceeding to the RNA extraction and RT-qPCR analysis of the autophagy genes. (**B**) Cells were grown for 24 h in the control medium (CTRL) supplemented or not with the indicated concentration of HF. Then, RNA extraction and RT-qPCR analysis of the atg genes were performed. Results are presented as the relative quotient (RQ) normalized to the *T* = 0 h time point (**A**) or the control condition (**B**). Data are presented as means ± SEM, *N* = 4. Conditions showing results statistically different from each other are presented using a different letter (*p* < 0.05, one-way ANOVA Tukey’s post-hoc test).

As mentioned previously, autophagy is an intracellular degradation process that is stimulated upon starvation conditions. This stimulation is controlled at different transcriptional, translational and post-translational levels. We first sought to determine the transcriptional time course of specific *atg* genes—namely, *lc3b*, *atg4*, *sqstm1* and *atg12* (Figure 3A).

**Figure 4:**
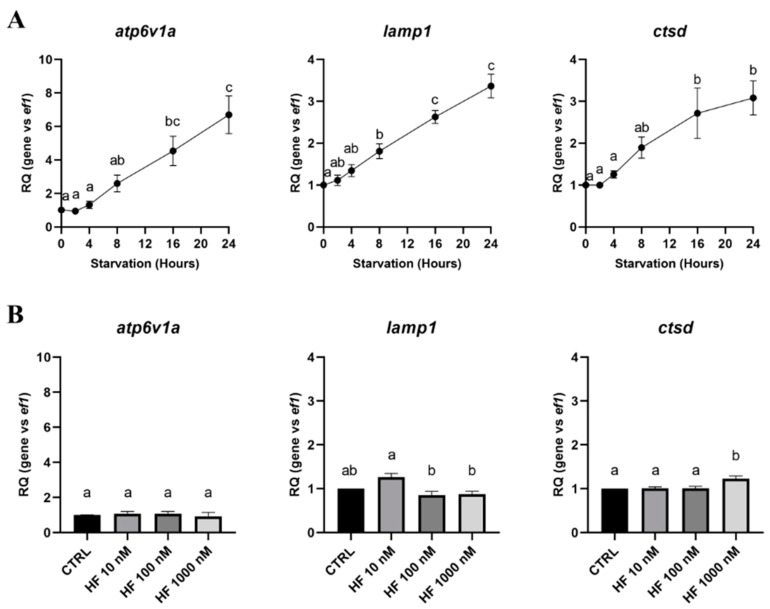
Expression of lysosomal markers induced by starvation and HF in RTH-149 cells. (**A**) Cells were starved during the indicated times prior to proceeding to the RNA extraction and RT-qPCR analysis of the lysosomal genes. (**B**) Cells were grown for 24 h in the control medium (CTRL) supplemented or not with the indicated concentration of HF. Then, RNA extraction and RT-qPCR analysis of the lysosomal genes were performed. Results are presented as the relative quotient (RQ) normalized to the *T* = 0 h time point (**A**) or the control condition (**B**). Data are presented as means ± SEM, *N* = 4. Conditions showing results statistically different from each other are presented using a different letter (*p* < 0.05, one-way ANOVA Tukey’s post-hoc test).

If all genes tested showed a significant increase following 24 h of starvation, the time courses were slightly different between each other. Indeed, *sqstm1* expression is the first to be significantly upregulated (8 h), while it takes 16 h of starvation to detect *lc3b* upregulation and 24 h for the *atg4* and *atg12* genes. Moreover, the treatments of the RTH-149 cells with HF (Figure 3B) showed that *lc3b*, *atg4* and *sqstm1,* but not *atg12*, were upregulated following the GCN2 pathway activation. Then, knowing that autophagy requires functional lysosomes to be completed, we also analyzed the expression of lysosomal markers upon starvation and the specific requirements of an active GCN2 pathway (Figure 4).

**Figure 5:**
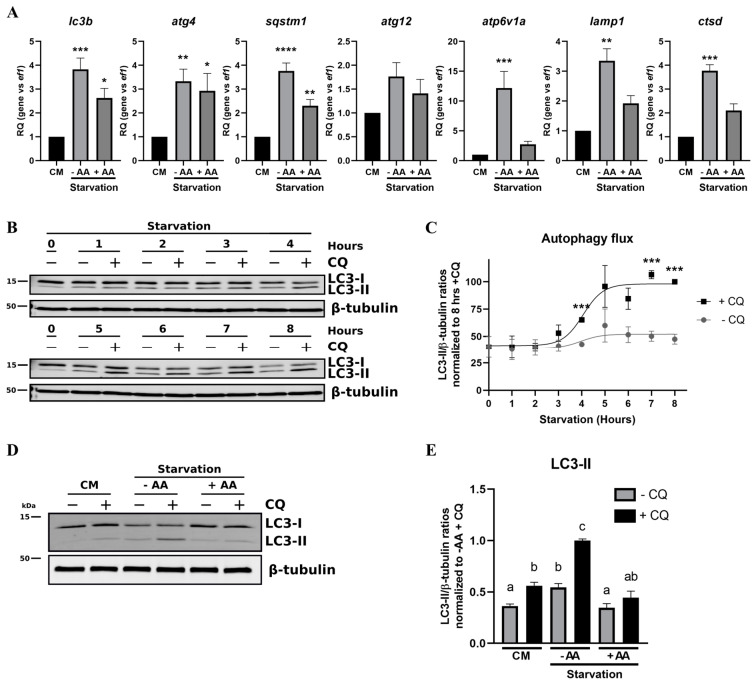
Amino acid-dependent regulation of starvation-induced autophagy in RTH-149 cells. (**A**) Cells were grown for 24 h in either complete medium (CM) or starvation medium supplemented (+AA) or not (−AA) with amino acids prior to proceeding to the RNA extraction and RT-qPCR analysis of the autophagy and lysosomal genes. Results are presented as the relative quotient (RQ) normalized to the CM. Data are presented as means ± SEM, *N* = 3. Conditions showing results statistically different from the control condition (CM) are presented as *: *p* < 0.05, **: *p* < 0.01, ***: *p* < 0.001 and ****: *p* < 0.0001, one-way ANOVA Dunnett’s post-hoc test. (**B**) Cells were starved for the indicated time prior to proceeding to the protein extraction and Western blot analysis using antibodies directed against LC3B and β-tubulin as a loading control. (**C**) Quantifications of the LC3-II and β-tubulin levels from the Western blots were performed using ImageJ. Results are presented as ratios of LC3-II on β-tubulin levels normalized to the 8 h of starvation with the CQ condition. Data are presented as mean values ± SEM, *N* = 3. For each time point, results were compared between +CQ vs. −CQ. Statistical differences are shown as ***: *p* < 0.001, Student’s *t*-test. (**D**) Cells were grown for 5 h in either complete medium (CM) or starvation medium supplemented (+AA) or not (−AA) with amino acids and 10 µM of CQ (−/+ CQ) prior to proceeding to the protein extraction and Western blot analysis using antibodies directed against LC3B and β-tubulin as a loading control. (**E**) Quantifications of the LC3-II and β-tubulin levels were made using ImageJ software. Results are presented as ratios of LC3-II on β-tubulin levels normalized to starvation without amino acids and with the CQ (−AA +CQ) condition. Data are presented as means ± SEM, *N* = 5. Conditions showing results statistically different from each other are presented using a different letter (*p* < 0.05, one-way ANOVA Tukey’s post-hoc test).

Whatever their final localization, such as the lysosomal membrane for the V-type proton ATPase catalytic subunit A (Atp6v1a) and the lysosome-associated membrane glycoprotein 1 (Lamp1) or the lysosomal lumen for cathepsin D (Ctsd), all the lysosomal markers tested showed a strong increase, with a maximum between 16 and 24 h of starvation, according to the gene considered (Figure 4A). However, we observed that the *atp6v1a*, *lamp1* and *ctsd* gene expressions were not, or very slightly, affected by HF-induced GCN2 activation. (Figure 4B). So, to better understand if amino acids control autophagy in RTH-149 cells, we therefore analyzed how autophagy and lysosomal genes were regulated in cells grown in the control or starvation conditions supplemented or not with amino acids (Figure 5A).

Interestingly, we noticed that, if starvation induced an upregulation of all the markers tested, the supplementation of the starvation medium with amino acids showed minor effects on the autophagy genes, while the lysosomal markers were no longer upregulated (Figure 5A). In light of these results, it appeared essential to assess, at the protein level, how the autophagy flux is induced in RTH-149 cells and how it is regulated by amino acids. Therefore, we first initiated a set of experiments to define the time point at which autophagy induction can be detected (Figure 5B). A Western blot analysis performed against the Lc3b protein revealed an accumulation of LC3-II in the presence of CQ from 4 h until 8 h of starvation (Figure 5C), suggesting that the autophagy flux is efficiently induced following at least 4 h of starvation. Based on these results, we then conducted experiments in which RTH-149 cells were grown for 5 h in control or starvation conditions supplemented or not with amino acids and CQ (Figure 5D,E). The LC3b protein detected by Western blot clearly showed that the accumulation of LC3-II observed upon starvation in the presence of CQ was considerably decreased when amino acids were added to the starvation medium. Quantifications of the LC3-II levels confirmed that the presence of amino acids in the starvation medium significantly restored an autophagy flux similar to a control condition. Altogether, these results demonstrate that the autophagy pathway is conserved and tightly regulated by amino acids in RTH-149 cells.

### 3.3. mTOR

**Figure 6:**
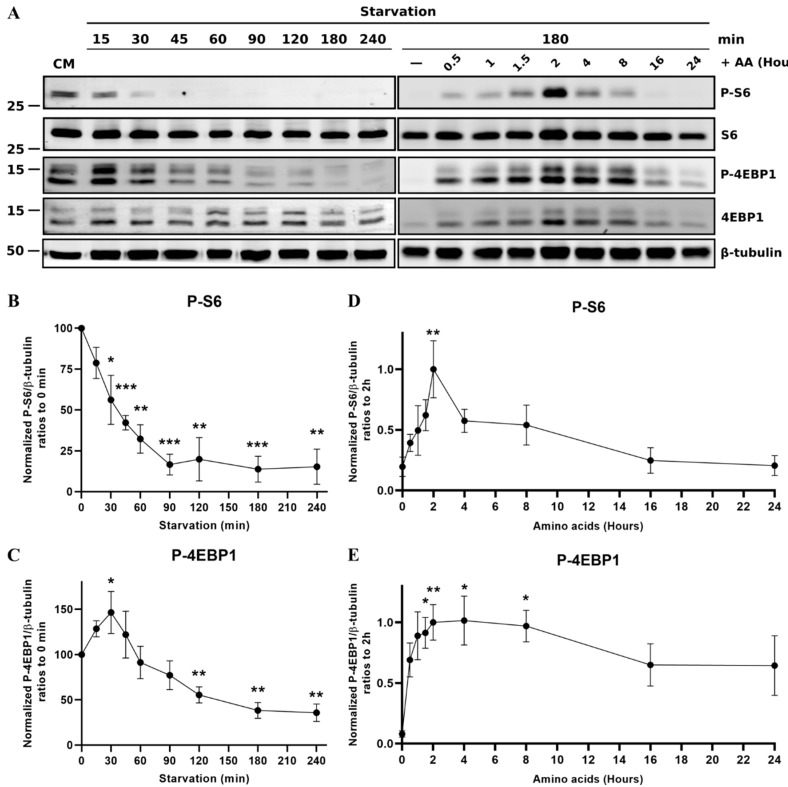
Regulation of the mechanistic Target Of Rapamycin (mTOR) pathway activity following starvation or stimulation with amino acids in RTH-149 cells. (**A**) Left panel: Cells were starved for the indicated time. Right panel: Cells were starved for 3 h prior to being treated using the starvation medium supplemented with amino acids for the indicated time. Following the protein extractions, Western blots were performed using antibodies directed against phospho-S6, total S6, phospho-4EBP1 and total 4EBP1 proteins and β-tubulin as a loading control. (**B**–**E**) Quantifications were performed using ImageJ software, and ratios between the indicated phosphorylated protein levels and β-tubulin were calculated prior to being normalized to *T* = 0 h (**B**,**C**) or *T* = 2 h (**D**,**E**). Data are presented as means ± SEM, *N* = 3. Conditions showing results statistically different from the 0 h time point are presented as *: *p* < 0.05, **: *p* < 0.01 and ***: *p* < 0.001, one-way ANOVA Dunnett’s post-hoc test.

The mTOR pathway is another major cellular pathway that is described to respond to amino acid availability. In contrast with the GCN2 and autophagy pathways, this pathway is inactivated upon starvation and activated in the presence of intracellular amino acids to keep cellular homeostasis. Thus, we sought to analyze the rate at which mTOR is inactivated upon starvation, as well as its specific kinetic of activation by amino acids by conducting a Western blot analysis directed against the phospho-4EBP1 and phospho-S6 proteins. For this, we used protein extracts from RTH-149 cells starved for different time points or starved prior to being stimulated back with amino acids for the indicated time (Figure 6).

If the dephosphorylation of S6 appeared to start following only 15 min of starvation to finally become undetectable after 60 min, surprisingly, the level of phospho-4EBP1 increased in the first instance before finally decreasing (Figure 6A, left panel). Quantifications of the dephosphorylation rates observed upon starvation for the S6 and 4EBP1 proteins revealed that, if the S6 phosphorylation levels continuously decreased to a minimum state observed following 90 min of starvation (Figure 6B), for unknown reasons, the 4EBP1 phosphorylation level significantly increased after 30 min of starvation prior to becoming significantly dephosphorylated after 2 h (Figure 6C). In light of these results, and to observe the activation of the mTOR by amino acids, the cells were first starved for 3 h, the starvation time required for S6 and 4EBP1 to display the lowest phosphorylation, prior to being stimulated with amino acids for the indicated time. This set of experiments allowed a fine detection of the phosphorylation induced by amino acid treatments of these targets (Figure 6A, right panel). Thus, signals of the phospho-S6 and phospho-4EBP1 proteins were detected early following amino acid stimulation (30 min), but quantifications revealed different kinetics between each target (Figure 6D,E). Indeed, S6 phosphorylation is significant and maximal only after 2 h of stimulation, while 4EBP1 becomes significantly faster and longer phosphorylated (from one hour and a half to 8 h of amino acid stimulation). According to these results, the following experimentations were conducted with the optimized protocol, meaning a starvation of cells for 3 h prior a stimulation with amino acids for 1.5 or 2 h.

**Figure 7:**
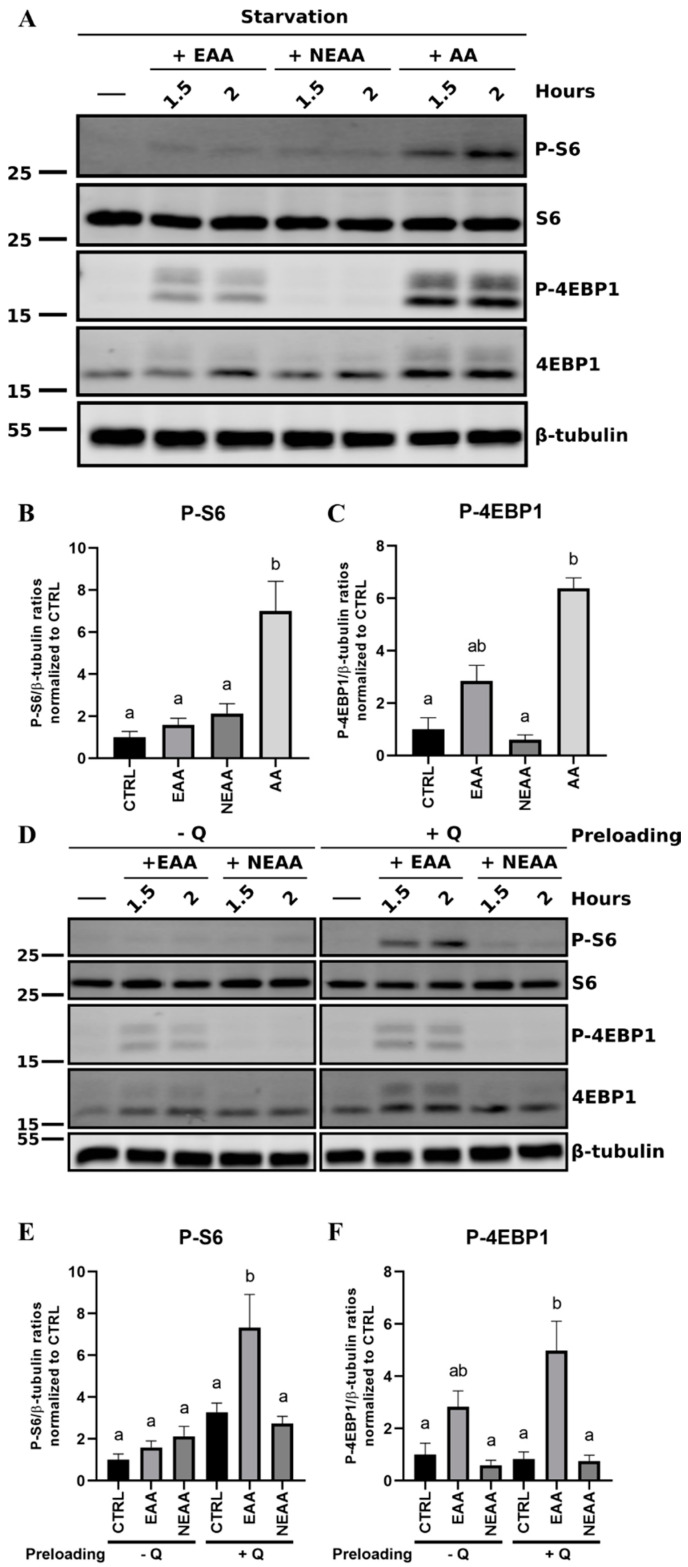
Regulation of essential and nonessential amino acids on mTOR activation in RTH-149 cells. (**A**) Cells were starved during 3 h prior to being treated with the starvation medium supplemented either with total (AA), essential (EAA) or nonessential amino acids (NEAA) during the indicated time. Following protein extractions, Western blots were performed using antibodies directed against phospho-S6, total S6, phospho-4EBP1 and total 4EBP1 proteins and β-tubulin as a loading control. (**B**,**C**) Quantifications were performed using ImageJ software, and ratios between the indicated phosphorylated protein levels and β-tubulin were calculated prior to being normalized to the CTRL condition at 2 h. Data are presented as means ± SEM, *N* = 3. Conditions showing results statistically different from each other are presented using a different letter (*p* < 0.05, one-way ANOVA Tukey’s post-hoc test). (**D**) Experiments were performed as in (**A**), except that cells were starved in the presence or not of glutamine (−/+Q) prior 1.5 or 2 h-treatments with EAA or NEAA. (**E**,**F**) Quantifications and analyses of 2 h-treatments were made as described in (**B**,**C**). Data are presented as means ± SEM, *N* = 3. Conditions showing results statistically different from each other are presented using a different letter (*p* < 0.05, one-way ANOVA Tukey’s post-hoc test).

Thereafter, we sought to evaluate how the mTOR could be specifically activated by two independent groups of amino acids: EAA (containing arginine) or nonessential amino acids (NEAA) supplemented with glutamine (Q) (Figure 7A). Astonishingly, when treated independently with these two pools of amino acids, no or low phosphorylation of S6 and 4EBP1 were detected compared to the phosphorylation levels observed and quantified with the total amino acid treatments (Figure 7B,C). Nonetheless, when the RTH-149 cells were preloaded with glutamine prior to repeating the stimulation with EAA or NEAA, the mTOR was only activated by EAA, as observed by a Western blot analysis (Figure 7D) and their respective quantifications (Figure 7E,F). Altogether, these results demonstrate that RTH-149 cells display a functional mTOR pathway that can sense the availability of amino acids.

## 4. Discussion

In recent years, the use of the primary cultures of myoblasts, adipocytes or hepatocytes increased significantly to study molecular mechanisms related to the nutrition of fish of agronomic interests [28,29,30,31,32,33,34,35]. Although this approach led to significant progress in the field, it also revealed its weaknesses. Indeed, beside that the society is more than ever pushing to restrict animal experimentations, primary cell cultures do not allow yet (or only to a very limited extent) a functional genomic analysis and, thus, limits our understandings of specific gene functions.

In the present study, we sought to investigate the usefulness of a RT cell line to address nutrition-related questions by focusing our work on three major pathways shown to regulate cell homeostasis through their regulation by amino acids. Our work clearly demonstrates that RTH-149 cells display functional GCN2, autophagy and mTOR pathways. Indeed, the results obtained clearly demonstrate that starvation can be sensed by RTH-149 cells, which then induce the activation of GCN2 and drive the expression of ISR-related genes in an amino acid-dependent manner. In detail, the results showed that a high concentration of HF (1000 nM) strongly upregulates *chop* but represses the induction of other ISR-related genes. This result corroborates previous findings from different species demonstrating that Chop overexpression contributes to a negative feedback loop responsible for attenuating the starvation-induced GCN2 response [36]. Furthermore, similarities in the regulation of the autophagy pathways were observed in RTH-149 cells compared to other species. For example, the regulations of *sqstm1*, *atg4* and *lc3b* observed in RTH-149 cells were already described in a mouse cell line to be induced following starvation in a GCN2/ATF4-dependent manner [37]. To the same extent, the kinetic of the starvation-induced autophagy measured in RTH-149 cells almost perfectly matches with the one measured in starved mouse embryonic fibroblast (MEF) cells [38]. Finally, the results obtained in RTH-149 cells related to the amino acid-induced mTOR activation are also consistent with those obtained in other species. Indeed, in RTH-149 cells, the mTOR appeared to be strongly activated by EAA when the cells were preloaded with glutamine. This observation would indicate that RTH-149 cells sense amino acids and activate the mTOR following mechanisms shared between trout, mice and human cell lines [39,40] Altogether, experiments conducted so far in RTH-149 cells tend to demonstrate that molecular mechanisms leading to GCN2, autophagy and mTOR activation have been conserved throughout evolution.

Nonetheless, if the results obtained in the RTH-149 cell line backup the main results observed in other species and, also, in previous studies performed in primary RT cell cultures [41,42,43], our data also highlights some particularities that RTH-149 cells display for these three pathways. For instance, we observed that *atg12* was not upregulated following the HF treatment, indicating that *atg12* is likely not a target of the GCN2 pathway in RTH-149 cells, while it is in MEF cells [37]. Furthermore, we observed that the overexpression of *atg* genes upon starvation was not repressed in the presence of amino acids in RTH-149 cells, in contrast to observations previously made in primary RT myoblasts [41]. To better understand these differences in the regulation of *atg* genes, future studies will have to be conducted to determine, for instance, if this could be related to the tissue from which the cell line derived or related to the transformation/immortalization process. Finally, despite the increase of phosphorylation of 4EBP1 observed in the earlier time points of starvation, which has not been reported so far in other species, we observed that the mTOR pathway reacts more slowly to starvation, or to a stimulation with amino acids, than the responses observed in other species. Indeed, when it takes less than 20 min for the mammalian mTOR pathway to be activated by amino acids [44], in RTH-149 cells, we observed a significant increase in the phosphorylation levels of S6 and 4EBP1 following 2 h of stimulation. On one hand, an explanation for this delayed response could be that, since rainbow trout have evolved to grow in cold water (24 °C or higher temperatures being lethal for trout), the whole process could be slowed down by the growing temperature routinely used for cell cultures (18 °C). On the other hand, we observed that the kinetics of the activation of the autophagy process in RTH-149 cells is comparable with those observed in mammalian cells. Altogether, these observations likely point to intrinsic specificities of the RTH-149 cells and, finally, draw the attention of future studies to the need to characterize the time responses of these pathways in other cell lines, or primary cell cultures, to avoid jumping to conclusions on the effects of a specific nutrient or stress. Moreover, the results obtained on the autophagy flux in starved RTH-149 cells demonstrated, one more time, the complexity of autophagy, which is regulated at multiple levels and by different stresses, and strengthened the need to follow guidelines to assess autophagy [22]. Indeed, if we observed that *atg* gene expressions were not regulated by the amino acid availability, the autophagic flux assay clearly showed a repression of the starvation-induced autophagy in the presence of amino acids. Independently, each result could lead to opposite conclusions, but, according to the literature, from different species previously mentioned and to the different time courses measured for the mTOR and autophagy in our study, this characterization of the autophagy response in RTH-149 cells likely supports the canonical model for which the mTOR regulates autophagy via the amino acid-sensing pathway.

Altogether, our study supports, for the first time, the use of RTH-149 cells in nutrition-related topics. Indeed, it presents a full set of protocols, and it identifies numbers of key molecular markers together with their specific time-dependent activations that could be of great interest for future experimentations aiming to better understand molecular mechanisms involved in, and controlling, fish nutrition. The use of RT cell lines, through nutritional approaches, will certainly help to develop new feed formulations using alternative protein sources rather than FM. For instance, vegetal proteins are well-described to lack some EAA (e.g., methionine, lysine and threonine) [45] that are required to be supplemented to the diet as free amino acids to cover, theoretically, the metabolic needs of fish. For instance, numerous studies previously performed in vivo evaluated if a supplementation of the missing amino acids could be effective on trout growth and, if so, at which level of supplementation [46]. Therefore, it is now well-described that EAA supplementation in plant-based diets prevent, but only partially, the growth retardation observed in fish fed with regular plant-based diets. From now on, new feedstuffs are being studied to replace FM, such as yeast- and insect-based diets, but, even if they theoretically display proteins well-balanced for EAA, the demonstration that their general amino acid profiles meet the metabolic fish needs is not yet confirmed. In this context, in vitro approaches using RT cell lines will address these questions, for instance, by mimicking the amino acid composition of the alternative protein source to grow cells and look at the cellular outcomes prior to proceeding to in vivo validations usually considered as being heavier and time-consuming.

Finally, considering the abundance of cell lines in the RT invitromatic, we believe that the use of cell lines to study fish nutrition and metabolism will be of great interest in the close future, especially with the recent advances in gene invalidation technics, such as CRISPR/cas9. Definitively, the combination of such approaches will therefore allow to understand deeper the relationships between nutrients and fish homeostasis and will certainly help to design new feed formulations for the development of an aquaculture that is sustainable and eco-responsible.

## Figures and Tables

**Table 1:** List of real-time quantitative PCR (RT-qPCR) primers used in this study.

Genes	Forward Primer	Reverse Primer
*asns*	5′-CTGCACACGGTCTGGAGCTG-3′	5′-GGATCTCGTCTGGGATCAGGTT-3′
*chop*	5′-CGACAATGTCCAACAACCTG-3′	5′-ACGAGGAGAACGAGGTGCTA-3′
*xbp1*	5′- TGCAACCAAGCCAATTCTTC -3′	5′-GCGAGAACTTCGTCTTCCAG-3′
*edem1*	5′-GAACATCCAAACGGGACAGT-3′	5′-TGAGAAGAGGGAGGGAGTCA-3′
*lc3b*	5′-GAAACAGTTTGACCTGCGTGAA-3′	5′-TCTCTCAATGATGACCGGAATCT-3′
*sqstm1*	5′-AGCCCACTGGGTATCGATGT-3′	5′-GGTCACGTGAGTCCATTCCT-3′
*atg4b*	5′-TATGCGCTTCCGAAAGTTGTC-3′	5′-CAGGATCGTTGGGGTTCTGC-3′
*atg12*	5′-GATGGAGGCCAATGAACAGC-3′	5′-GCGTTTGAACTGAAAAGGGCTAA-3′
*atp6v1a*	5′-CTGTTTAATTTCTGAAGATCTAGCC-3′	5′-GATCTCTCCCACCAGCTCAC-3′
*ctsd*	5′-CAGACATCGCCTGCTTGCTT-3′	5′-CAGGATGCCGTCAAACTTCG-3′
*ef1*	5′-TCCTCTTGGTCGTTTCGCTG-3′	5′-ACCCGAGGGACATCCTGTG-3′
